# CuidaTXT: A Text Message Dementia-Caregiver Intervention for Latinos

**DOI:** 10.1093/geroni/igaa057.2777

**Published:** 2020-12-16

**Authors:** Jaime Perales Puchalt

**Affiliations:** University of Kansas, Fairway, Kansas, United States

## Abstract

Latino family caregivers of people with dementia have low access to caregiver support. Text messaging holds potential to dramatically enhance the reach of caregiver support interventions among Latinos. This presentation will describe the CuidaTXT Project, with a special emphasis on approach to recruitment and community engagement to achieve the objectives of designing and testing the first dementia caregiver-support text message intervention for Latinos. Based on the Stress Process Framework, CuidaTXT incorporates social support and coping components including AD education, problem-solving skills training, social network support, care management and referral to community resources via tailored two-way messaging. Engagement in CuidaTXT benefited from multi-source recruitment efforts in the Latino community-network built over a three-year period. The network is comprised of senior, religious and community centers, the local media, clinics, a Latino registry and a dementia health navigation service. This presentation will describe processes for assembling and engaging the network for CuidaTXT.

